# Effects of Cognitive Remediation on Cognition, Metacognition, and Social Cognition in Patients With Schizophrenia

**DOI:** 10.3389/fpsyt.2021.649737

**Published:** 2021-07-09

**Authors:** Cristiana Montemagni, Elisa Del Favero, Cecilia Riccardi, Laura Canta, Mario Toye, Enrico Zanalda, Paola Rocca

**Affiliations:** ^1^Department of Neuroscience “Rita Levi Montalcini”, Azienda Ospedaliera Universitaria (AOU) Città della Salute e della Scienza, University of Turin, Turin, Italy; ^2^Department of Mental Health, Azianda Sanitaria Locale (ASL) TO3 & Azienda Ospedaliera Universitaria (AOU) San Luigi Gonzaga, Orbassano, Italy

**Keywords:** schizophrenia, cognitive remediation, metacognition, social cognition, cognition

## Abstract

We aimed to evaluate in a sample of outpatients with schizophrenia (SCZ) the effectiveness of a cognitive remediation (CR) program (through the use of the Cogpack software) [computer-assisted CR (CACR)] in addition to standard therapy on cognitive outcomes as compared with that in a control active group (CAG) and to highlight a possible effect on social cognition (SC), metacognition, symptomatology, and real-world functioning. Of the 66 subjects enrolled, 33 were allocated to CACR and 33 to the CAG. Twenty-three patients in the CACR group and 25 subjects in the CAG completed at least 80% of the 48 prescribed CACR sessions, performed twice a week, for a total of 24 weeks of treatment. A significant time × group interaction was evident, suggesting that patients undergoing CACR intervention improved in specific metacognitive sub-functions (understanding others' mind and mastery), some cognitive domains (verbal learning processing speed, visual learning, reasoning, and problem solving) (h^2^ = 0.126), depressive symptoms, SC, awareness of symptoms, and real-world functioning domains (community activities and interpersonal relationships) more significantly than did patients undergoing CAG. The most noticeable differential improvement between the two groups was detected in two metacognitive sub-functions (understanding others' mind and mastery), in verbal learning, in interpersonal relationship, and in depressive symptomatology, achieving large effect sizes. These are encouraging findings in support of the possible integration of CACR in rehabilitation practice in the Italian mental health services.

## Introduction

Cognitive deficits represent one of schizophrenia's (SCZ's) core features since they affect patients' functioning and are related to high levels of functional disability (1–4).

Moreover, cognitive impairment attenuates the potential benefit of rehabilitation programs, such as supported employment and social skills training-, even when high-quality rehabilitation is provided (5–7).

Since cognitive deficits of patients with SCZ respond only moderately to pharmacotherapy (8), cognitive remediation (CR) techniques have received an increasing development in the last 15 years.

At the Cognitive Remediation Experts Workshop (Florence, Italy, April 2010), CR has been defined as “a behavioral training based intervention that aims to improve cognitive processes (attention, memory, executive function, social cognition, or metacognition) with the goal of durability and generalization” (9).

It has proved to be an evidence-based approach for ameliorating cognitive impairment in SCZ as confirmed in different meta-analytic studies (9–12), which show significant effect-size impact on cognition (0.45) and daily functioning (0.36) (9, 10).

More specifically, Wykes et al. (9) found that cognitive outcomes improve with both drill-and-practice and strategic training; McGurk et al. (10) corroborated that drill or practice training alone has a larger effect on memory and verbal learning, when associated with strategic training. Lastly, Grynszpan et al. (11) proved smaller effect sizes on verbal memory, working memory, attention/vigilance, and speed of processing and a significant medium effect size for social cognition (SC), while general cognition provides positive results. Interestingly, it appears that effects of CR programs are “non-specific”; targeted cognitive domains did not reach higher improvement than those that were not specifically targeted.

Computer-assisted CR (CACR) effects on cognition have been widely analyzed in two recent meta-analyses. The first one (13) includes 24 studies specifically centered on computerized drill-and-practice training. This study demonstrated that, compared with a control condition, specific training obtains better results on attention, working memory, and positive and depressive symptoms. Furthermore, even if only marginally significant, small-to-moderate effects were noticed for verbal fluency, processing speed, memory, and verbal and visual learning. No evidence was found sustaining improvement in general condition, reasoning and problem solving, SC, and functional outcomes. The second one (14) evaluates CR treatment effect on cognitive, functional, and clinical outcomes in patients with SCZ from 67 studies and found significant improvements with small-to-moderate effect sizes in CR treatment on all of the three outcome domains and suggests that cognitive gains trigger improvement in both clinical symptoms and psychosocial functioning.

Thus, some discrepancies across studies about SCZ (15) were noticed regarding the improvement in community functioning and SC following CR, while an improvement in cognition seems to be commonly reported.

Therefore, in light of these considerations, the objective of the present study was to evaluate, in a sample of outpatients with SCZ, the effectiveness of a CR program (through the use of the Cogpack software) in addition to standard therapy on cognitive outcomes compared with a control active group (CAG) and to evaluate if there is any impact on SC, metacognition, symptomatology, and real-world functioning.

## Materials and Methods

### Subjects

The present study was conducted at the Department of Neuroscience “Rita Levi Montalcini,” University of Turin; Department of Neuroscience and Mental Health, Psychiatry Unit, AOU Città della Salute e della Scienza, Turin, Italy' and the Department of Mental Health, ASL TO3, Italy, between October 2017 and December 2019.

The trial was carried out in accordance with the Declaration of Helsinki of 1995 (as revised in Edinburgh in 2000) and was approved by the Local Ethical Committee on September 27, 2018 (Prot. N 00958893).

#### Inclusion Criteria

Patients, initially evaluated by a psychiatrist if they met the DSM-5 criteria for the diagnosis of SCZ, were subsequently visited by our research group. The patients examined were aged between 18 and 65 years. The study was conducted on a sample of outpatients with diagnosis of SCZ in a stable phase of illness.

#### Exclusion Criteria

The exclusion criteria were the following: (a) co-presence of a diagnosis of intellectual disability and learning disabilities; and (b) hospitalization in psychiatric facilities in the 6 months prior to evaluation and significant change of antipsychotic medications during the previous 3 months (according to clinical judgment). Participants were recruited through referrals from attending psychiatrists or clinical staff at the psychiatric medical facilities where the study was conducted.

#### Participants

**Figure 1:**
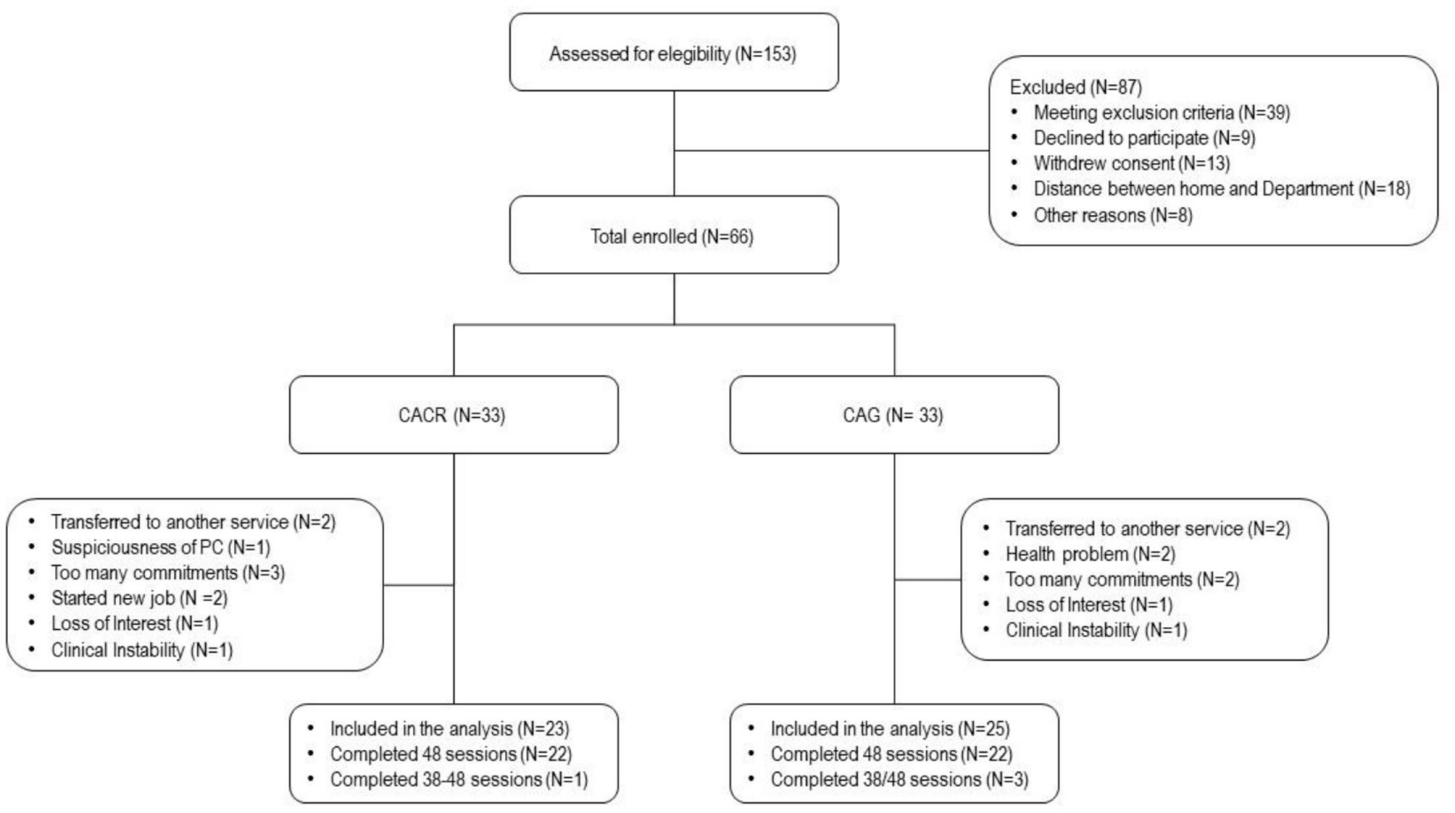
Flow diagram of assessment for the eligibility in the two groups.

Of the 153 patients with SCZ attending the two sites, only a minority was approached for the study. Finally, 66 patients were invited to participate. Details of factors contributing to reduction of the number of potential participants are given in Figure 1.

Written informed consent was obtained from all subjects after a complete description of the study.

All patients were submitted to standard care provided in community mental health centers in Italy (pharmacological treatment, clinical monitoring at least on a monthly basis, and home care when required).

### Interventions

All patients recruited in the trial were allocated to one of the two interventions, CACR (Cogpack), and CAG. In this trial, patients were considered completers if they attended at least 80% of the planned sessions.

When a participant decided not to continue or stopped the program session for at least 2 weeks or presented clinical relapse, the participant was considered to have dropped out. Reasons for discontinuation were registered.

### Control Active Group

This condition was designed to control for non-specific treatment effects. It specified an equal number of one-on-one computer sessions with the same trainers who conducted the CACR sessions, using the same schedule as the treatment arm: 2-h biweekly sessions. It offered supportive trainer interactions and matched experience with computers and varied computer activities. Control activities were selected for game-like properties and low cognitive demand. Participants in this condition did not receive problem-solving training or guided practice on the exercises used in the remediation condition.

### Cognitive Remediation Group

The CACR group received 48 sessions computerized rehabilitation using Cogpack software, performed twice a week, for a total of 24 weeks of treatment, in addition to standard therapy. CACR characteristics are described in another paper (16).

Patients were evaluated using a semi-structured interview to assess demographic and clinical features.

### Psychiatric Assessment

All subjects were evaluated at baseline (T0), after 3 months (T1), and after 6 months (T2).

Patients were assessed by two psychiatrists (ED and CR). Neuropsychological tests were administered by a trained psychologist (LC) who was unaware of the patients' clinical characteristics or the results of the psychiatric rating scales. The battery was administered and scored according to the established procedures for each test the day after the psychiatric assessment. The raters were all masked with regard to the patient's treatment assignment, and patients were instructed not to reveal their treatment to the investigators. The interviewing psychiatrists were never members of the patients' treating team.

#### Clinical Assessment

The Positive and Negative Syndrome Scale (PANSS) (17) contains 30 items rated on 1 (absent) to 7 (extreme) scales. Three symptom domains are included in the scale: positive symptoms (seven items), negative symptoms (seven items), and general psychopathology (16 items). Symptoms are typically rated for the week immediately preceding the interview.

Depressive symptoms were evaluated using the Calgary Depression Scale for Schizophrenia (CDSS) (18).

The Scale to assess the Unawareness of Mental Disorder (SUMD) was used to assess illness insight (19). For the purpose of the present research, data analysis was primarily focused on current awareness and attribution of symptoms. All the scores are located in a Likert-type scale from 1 (good insight) to 5 (no insight). The insight of the patients was categorized as generally “preserved” or “impaired” on a threshold mean score of ≤ 3.0.

#### Cognitive Assessment

The MATRICS Consensus Cognitive Battery (MCCB) (20) consists of 10 measurement tools for recording the six neurocognitive areas and the social–cognitive domain of emotion perception. The six neurocognitive measures derive from scores of 10 cognitive measures: processing speed (*Trail Making Test Part A*; *Brief Assessment of Cognition in Schizophrenia*: symbol coding; *Category Fluency Test*: animal naming), attention/vigilance (*Continuous Performance Test*: identical pairs), working memory (*Wechsler Memory Scale*: spatial span subset; *Letter Number Span Test*), verbal learning (refers to immediate verbal memory, *Hopkins Verbal Learning Test (HVLT)-Revised*, immediate recall), visual learning (refers to immediate visual memory, *Brief Visuospatial Memory Test-Revised*), reasoning, and problem solving (*Neuropsychological Assessment Battery* (NAB), mazes subtest). The social–cognitive domain is assessed by the *Mayer–Salovey–Caruso Emotional Intelligence Test* (MSCEIT) (21), managing emotion section.

The Metacognition Assessment Scale (MAS) (22) was developed to quantitatively assess synthetic aspects of metacognition. MAS-A is a modification of the original scale made in 2004 that contains four scales that pertain to different foci of metacognitive acts: *Self Reflectivity*, or the comprehension of one's own mental states; *Understanding of others*' *minds*, or the comprehension of other individuals' mental states; *Decentration*, which is the ability to see the world as existing with others having independent motives; and *Mastery*, which is the ability to use one's mental states to implement effective action strategies in order to accomplish cognitive tasks or cope with psychological distress.

#### Real-World Functioning Assessment

The Specific Levels of Functioning Scale (SLOF) (23–25) assesses the patient's current functioning and behavior across the following six domains: physical functioning, personal care skills, interpersonal relationships, social acceptability, activities of community living, and work skills. The rate of each questions is included between 0 (the lowest level of functioning evaluated) and 5 (the maximum level of functioning evaluated) on a 5-point Likert scale, and the exact time frame that the survey attempts to assess is unspecified.

### Research Design and Data Analysis

Analyses were planned in two stages.

In stage 1, we performed chi-square tests for categorical variables and the univariate analysis of variance (one-way ANOVA) for continuous variables, to examine whether the two groups differed in baseline demographic, clinical, neuropsychological, and functional variables. *p*-Values < 0.0083 (0.05/6) were considered significant.

In stage 2, pre-to-post treatment changes were analyzed with repeated-measures ANOVA entering neurocognitive, metacognitive, and functioning score subtests as the dependent variables; time as the fixed factor (with the three levels T0, T1, and T2); and treatment group as the independent variable. The significance of *F* test for group × time interaction will indicate if there are significant differences in outcomes between the two groups.

Size effect was assessed with partial η^2^. Values ranging between 0.01 and 0.06 were considered as small effect, values ranging between 0.07 and 0.14 were considered as medium effect, and values higher than 0.14 were considered as large effect (26).

The researchers ensured that there were no significant outliers in the related groups.

Statistical analyses were conducted using Statistical Package for the Social Sciences, SPSS, version 25 for Windows (SPSS, Chicago, IL, USA).

## Results

A total of 66 subjects were enrolled and provided consent to participate in the study. Thirty-three of these patients were allocated to CACR and 33 to the CAG. Ten patients dropped out from the CACR group and eight from the CAG. Therefore, the final sample included 23 patients in the CACR group and 25 subjects in the CAG. No statistical differences were found between dropouts and completers in both groups (Tables 1, 2).

**Table 1:** Differences between drop-outs and CAG group completers.

**Variables**	**Condition**		**SD**		**F/X^**2**^**	***p*-value**
	**CAG**	**Drop**	**CAG**	**Drop**		
Age	42.72	41.75	8.473	4.432	0.095	0.760
Gender (*male*)*	10	2			0.589	0.443
Employment (*unemployed*)*	8	1			1.162	0.281
Schooling (*years*)	11.08	12.63	3.82	2.875	1.100	0.302
Age of onset	25.68	21.13	7.5	3.441	2.729	0.109
PANSS-P	11.83	12.63	2.98	1.085	1.056	0.312
PANSS-N	22.0	24.13	7.12	1.856	0.602	0.444
PANSS-G	36.08	39.75	9.98	2.455	0.928	0.343
PANSS total	72.96	77.25	17.95	4.705	0.385	0.539
MAS self-reflectivity	4.52	6.13	2.35	0.459	3.346	0.081
MAS others' mind	3.76	3.13	2.03	0.479	0.680	0.416
MAS decentration	0.4	1.00	0.95	0.378	2.255	0.143
MAS mastery	2.92	2.88	2.71	0.125	0.030	0.863
MAS total	11.6	11.13	6.99	1.575	0.032	0.859
CDSS	1.8	1.75	2.04	0.526	0.004	0.950
Verbal memory	33.36	36.00	8.64	2.260	2.800	0.104
Reasoning/probl. solving	32.28	33.75	4.71	3.614	0.32	0.575
Visual memory	35.92	34.75	14.07	5.130	0.041	0.840
Attention/vigilance	28.36	27.63	9.03	1.456	0.049	0.827
SC	27.4	30.25	10.74	3.858	0.424	0.520
Processing speed	24.48	26.00	9.99	2.87	0.165	0.688
Working memory	29.24	36.00	10.76	2.260	2.800	0.104
SLOF physic func	24.6	24.63	1.04	0.183	0.004	0.949
SLOF personal care	32.76	34.25	2.78	0.126	2.085	0.159
SLOF Int. relation	22.12	20.13	4.3	1.716	1.231	0.276
SLOF social accept	31.44	32.50	4.12	0.732	0.482	0.493
SLOF activities	47.76	43.25	5.67	2.448	3.449	0.073
SLOF work skills	19.64	18.75	5.37	2.651	0.137	0.714
SLOF total	179.12	175.75	17.36	6.475	2.926	0.097
SUMD awareness	3.22	3,016	0.65	0.269	0.559	0.460
SUMD attribution	3.35	3.740	0.78	0.329	1.391	0.247

PANSS-P, Positive and Negative Syndrome Scale, positive symptoms; PANSS-N, Positive and Negative Syndrome Scale, negative symptoms; PANSS-G, Positive and Negative Syndrome Scale, general, psychopathology; PANSS Total, Positive and Negative Syndrome Scale, total; MAS Self-reflectivity, Metacognition Assessment Scale, self-reflectivity; MAS Others' mind, Metacognition Assessment Scale, understanding of others' mind; MAS Decentration, Metacognition Assessment Scale, decentration; MAS Mastery, Metacognition Assessment Scale, mastery; MAS Total, Metacognition Assessment Scale, total; CDSS, Calgary Depression Symptoms Scale; Verbal memory, MCCB, verbal memory; Reasoning/Probl. solving, MCCB, reasoning and problem solving; Visual memory, MCCB, visual memory; Attention/Vigilance, MCCB, attention/vigilance; SC, MCCB, social cognition; Processing speed, MCCB, processing speed; Working memory, MCCB, working memory; SLOF Physical func, Specific Levels of Functioning Scale, physical functioning; SLOF Personal care, Specific Levels of Functioning Scale, personal care skills; SLOF Int. relation, Specific Levels of Functioning Scale, interpersonal relationships; SLOF Social accept, Specific Levels of Functioning Scale, social acceptability; SLOF Activities, Specific Levels of Functioning Scale, activities of community living; SLOF Work skills, Specific Levels of Functioning Scale, work skills; SLOF Int. relation, Specific Levels of Functioning Scale, total; SUMD Awareness, Scale to Assess Unawareness in Mental Disorder, awareness; SUMD Attribution, Scale to Assess Unawareness in Mental Disorder, attribution; CAG, control active group. *Dichotomous variable.

**Table 2:** Differences between drop-outs and CACR group completers.

**Variables**	**Condition**		**SD**		**F/X^**2**^**	***p*-value**
	**Drop**	**CACR**	**Drop**	**CACR**		
Age	33.20	31.24	3.155	9.34	0.000	0.985
Gender (*male*)*	5	18			2.636	0.104
Employment (*unemployed*)*	5	6			1.793	0.181
Schooling (*years*)	11.40	12.88	4.377	2.49	0.858	0.356
Age of onset	24.60	23.32	1.857	4.81	0.254	0.618
PANSS-P	14.50	15.24	2.729	6.93	0.457	0.504
PANSS-N	23.60	23.87	2.554	5.75	0.054	0.818
PANSS-G	36.70	37.39	3.927	9.27	0.021	0.887
PANSS total	73.90	73.53	7.287	15.54	0.026	0.874
MAS self-reflectivity	5.80	6.7	0.573	2.08	0.935	0.341
MAS others' mind	3.00	3.78	0.615	1.98	0.459	0.503
MAS decentration	0.30	1.26	0.300	1.25	3.952	0.056
MAS mastery	1.50	3.39	0.872	2.78	1.987	0.179
MAS total	15.90	15.13	1.622	6.59	0.798	0.378
CDSS	1.10	2.78	0.379	3.37	3.653	0.065
Verbal memory	33.20	37.35	1.806	9	1.128	0.297
Reasoning/probl. solving	31.50	34.43	1.293	10	0.155	0.697
Visual memory	35.40	37.22	2.754	15.78	0.153	0.698
Attention/vigilance	27.60	34.83	9.03	2.980	4.094	0.053
SC	34.20	33.81	3.623	12.53	0.158	0.676
Processing speed	22.50	26.91	2.786	7.72	0.529	0.473
Working memory	30.90	33.3	3.659	8.57	0.057	0.812
SLOF physic func	24.70	24.63	0.153	0.87	2.301	0.140
SLOF personal care	32.70	32.65	0.978	3.3	0.271	0.607
SLOF Int. relation	20.30	20.96	1.484	5.79	0.005	0.945
SLOF social accept.	33.136	34.65	0.194	0.78	1.693	0.203
SLOF activities	45.30	44.7	1.898	6.87	1.061	0.807
SLOF work skills	18.80	19.74	1.526	6.37	0.044	0.836
SLOF total	173.50	177.7	4.679	19.25	0.223	0.640
SUMD awareness	3.136	2.74	0.194	0.93	3.847	0.059
SUMD attribution	3.332	3.03	0.268	1.13	1.377	0.250

PANSS-P, Positive and Negative Syndrome Scale, positive symptoms; PANSS-N, Positive and Negative Syndrome Scale, negative symptoms; PANSS-G, Positive and Negative Syndrome Scale, general, psychopathology; PANSS Total, Positive and Negative Syndrome Scale, total; MAS Self-reflectivity, Metacognition Assessment Scale, self-reflectivity; MAS Others' mind, Metacognition Assessment Scale, understanding of others' mind; MAS Decentration, Metacognition Assessment Scale, decentration; MAS Mastery, Metacognition Assessment Scale, mastery; MAS Total, Metacognition Assessment Scale, total; CDSS, Calgary Depression Symptoms Scale; Verbal memory, MCCB, verbal memory; Reasoning/Probl. solving, MCCB, reasoning and problem solving; Visual memory, MCCB, visual memory; Attention/Vigilance, MCCB, attention/vigilance; SC, MCCB, social cognition; Processing speed, MCCB, processing speed; Working memory, MCCB, working memory; SLOF Physical func, Specific Levels of Functioning Scale, physical functioning; SLOF Personal care, Specific Levels of Functioning Scale, personal care skills; SLOF Int. relation, Specific Levels of Functioning Scale, interpersonal relationships; SLOF Social accept, Specific Levels of Functioning Scale, social acceptability; SLOF Activities, Specific Levels of Functioning Scale, activities of community living; SLOF Work skills, Specific Levels of Functioning Scale, work skills; SLOF Int. relation, Specific Levels of Functioning Scale, total; SUMD Awareness, Scale to Assess Unawareness in Mental Disorder, awareness; SUMD Attribution, Scale to Assess Unawareness in Mental Disorder, attribution; CAG, control active group; CACR, computer-assisted cognitive remediation. *Dichotomous variable.

The flow diagram in Figure 1 shows dropout reasons in the two groups.

Statistical analyses were performed on patients who completed the sessions of treatment.

All the demographical, clinical, neuropsychological, metacognitive, and functioning characteristics of the sample are reported in Table 3.

**Table 3:** Demographical, clinical, neuropsychological, metacognitive and functioning characteristics of the sample.

**Variables**	**Condition**		**SD**		**F/X^**2**^**	***p*-value**
	**CAG**	**CACR**	**CAG**	**CACR**		
Age	42.72	31.24	8.47	9.34	25.1	<0.001
Gender (*male*)*	10	18			1.79	0.181
Employment (*unemployed*)*	8	6			0.20	0.653
Schooling (*years*)	11.08	12.88	3.82	2.49	5.37	0.02
Age of onset	25.68	23.32	7.5	4.81	2.44	0.12
PANSS-P	11.83	15.24	2.98	6.93	2.07	0.16
PANSS-N	22.0	23.87	7.12	5.75	0.4	0.53
PANSS-G	36.08	37.39	9.98	9.27	0.33	0.57
PANSS total	72.96	73.53	17.95	15.54	0.02	0.9
MAS self-reflectivity	4.52	6.7	2.35	2.08	15.02	<0.001
MAS others' mind	3.76	3.78	2.03	1.98	0.21	0.65
MAS decentration	0.4	1.26	0.95	1.25	11.1	0.001
MAS mastery	2.92	3.39	2.71	2.78	0.18	0.67
MAS total	11.6	15.13	6.99	6.59	5.03	0.03
CDSS	1.8	2.78	2.04	3.37	3.69	0.059
Verbal memory	33.36	37.35	8.64	9	2.99	0.09
Reasoning/Probl. solving	32.28	34.43	4.71	10	2.15	0.15
Visual memory	35.92	37.22	14.07	15.78	1.18	0.28
Attention/vigilance	28.36	34.83	9.03	10.57	5.41	0.02
SC	27.4	33.81	10.74	12.53	7.85	0.01
Processing speed	24.48	26.91	9.99	7.72	0.96	0.33
Working memory	29.24	33.3	10.76	8.57	3.75	0.06
SLOF physic func	24.6	24.63	1.04	0.87	0.01	0.92
SLOF personal care	32.76	32.65	2.78	3.3	0.04	0.85
SLOF Int. relation	22.12	20.96	4.3	5.79	1.0	0.32
SLOF social accept	31.44	34.65	4.12	0.78	8.53	0.01
SLOF activities	47.76	44.7	5.67	6.87	2.1	0.15
SLOF work skills	19.64	19.74	5.37	6.37	0.19	0.67
SLOF total	179.12	177.7	17.36	19.25	0.14	0.71
SUMD awareness	3.22	2.74	0.65	0.93	10.839	0.002
SUMD attribution	3.35	3.03	0.78	1.13	3.328	0.073

PANSS-P, Positive and Negative Syndrome Scale, positive symptoms; PANSS-N, Positive and Negative Syndrome Scale, negative symptoms; PANSS-G, Positive and Negative Syndrome Scale, general, psychopathology; PANSS Total, Positive and Negative Syndrome Scale, total; MAS Self-reflectivity, Metacognition Assessment Scale, self-reflectivity; MAS Others' mind, Metacognition Assessment Scale, understanding of others' mind; MAS Decentration, Metacognition Assessment Scale, decentration; MAS Mastery, Metacognition Assessment Scale, mastery; MAS Total, Metacognition Assessment Scale, total; CDSS, Calgary Depression Symptoms Scale; Verbal memory, MCCB, verbal memory; Reasoning/Probl. solving, MCCB, reasoning and problem solving; Visual memory, MCCB, visual memory; Attention/Vigilance, MCCB, attention/vigilance; SC, MCCB, social cognition; Processing speed, MCCB, processing speed; Working memory, MCCB, working memory; SLOF Physical func, Specific Levels of Functioning Scale, physical functioning; SLOF Personal care, Specific Levels of Functioning Scale, personal care skills; SLOF Int. relation, Specific Levels of Functioning Scale, interpersonal relationships; SLOF Social accept, Specific Levels of Functioning Scale, social acceptability; SLOF Activities, Specific Levels of Functioning Scale, activities of community living; SLOF Work skills, Specific Levels of Functioning Scale, work skills; SLOF Int. relation, Specific Levels of Functioning Scale, total; SUMD Awareness, Scale to Assess Unawareness in Mental Disorder, awareness; SUMD Attribution, Scale to Assess Unawareness in Mental Disorder, attribution; CAG, control active group; CACR, computer-assisted cognitive remediation. *Dichotomous variable.

No statistically differences in gender distribution, clinical, and cognitive variables, and real-world functioning emerged between the two groups, except for age, MAS self-reflectivity, MAS decentration, as patients in the CACR group were younger, and had a higher level of metacognition (self-reflectivity and decentration) than those in the control group.

A significant improvement over trial duration **(**within-group effect) was observed for both treatments in verbal learning, MAS self-reflectivity, and MAS total score.

Moreover, patients in the CACR group demonstrated significantly greater improvements over 6 months than the control group (time × treatment interaction), with effect sizes ranging from medium to high levels for MAS others' mind (h^2^ = 0.436), verbal learning (h^2^ = 0.321), SLOF interpersonal relationship (h^2^ = 0.224), depressive symptoms (h^2^ = 0.215), SC (h^2^ = 0.183), MAS mastery (η^2^ = 0.178), processing speed (η^2^ = 0.143), visual learning (h^2^ = 0.131), reasoning and problem solving (h^2^ = 0.126), SLOF activities (h^2^ = 0.105), and awareness of symptoms (h^2^ = 0.063).

Table 4 presents scores for the neuropsychological, clinical, and functional outcomes at baseline and post-intervention.

**Table 4:** Scores for the neuropsychological, clinical, and functional outcomes at baseline and post-intervention.

**Variables**	**T**	**CAG (*n* = 25)**	**CACR (*n* = 23)**	**Source**	***F***	***p*-value**	**η2 partial**
		**M ± SD**	**M ± SD**				
PANSS-P	T0	11.83 ± , 2.98	15.24 ± 6.92	Group	1.639	0.209	0.047
	T1	11.26 ± 3.57	12.00 ± 6.92	Time	4.012	0.053	0.108
	T2	12.13 ± 4.61	12.00 ± 3.92	G * T	0.602	0.443	0.018
PANSS-N	T0	22 ± 7.12	23.87 ± 5.75	Group	0.009	0.252	0.040
	T1	21.52 ± 6.48	20.78 ± 4.5	Time	0.688	0.413	0.020
	T2	21.52 ± 6.48	19.61 ± 4.53	G * T	2.817	0.103	0.079
PANSS-G	T0	36.08 ± 9.98	37.39 ± 9.27	Group	0.779	0.384	0.023
	T1	33.88 ± 11.96	32.70 ± 7.23	Time	0.803	0.377	0.024
	T2	33.88 ± 11.96	31.43 ± 7.14	G * T	1.007	0.323	0.030
MAS self-reflectivity	T0	4.52 ± 2.35	6.7 ± 2.08	Group	20.839	0.000	0.387
	T1	4.24 ± 2.11	7.0 ± 1.83	Time	6.641	0.002	0.161
	T2	4.64 ± 2.1	7.35 ± 1.27	G * T	2.212	0.146	0.063
MAS others' mind	T0	3.76 ± 2.03	3.78 ± 1.98	Group	4.421	0.043	0.118
	T1	3.8 ± 1.76	4.09 ± 1.89	Time	3.947	0.055	0.107
	T2	3.76 ± 1.83	4.83 ± 1.7	G * T	25.467	0.000	0.436
MAS decentrat	T0	0.4 ± 0.96	1.26 ± 1.25	Group	1.024	0.000	0.487
	T1	0.52 ± 0.87	1.35 ± 1.19	Time	1.053	0.312	0.031
	T2	0.68 ± 1.14	1.61 ± 1.08	G * T	0.009	0.926	0.000
MAS mastery	T0	2.92 ± 2.71	3.39 ± 2.78	Group	0.095	0.760	0.003
	T1	2.56 ± 1.94	3.35 ± 2.41	Time	1.788	0.190	0.051
	T2	2.88 ± 2.28	4.39 ± 1.99	G * T	7.148	0.012	0.178
MAS total	T0	11.6 ± 6.99	15.13 ± 6.59	Group	8.79	0.005	0.16
	T1	11.12 ± 5.55	15.83 ± 5.58	Time	5.89	0.004	0.114
	T2	11.96 ± 6.64	18.22 ± 4.64	G * T	2.96	0.057	0.06
CDSS	T0	1.8 ± 2.04	2.78 ± 3.37	Group	0.505	0.482	0.015
	T1	3.68 ± 3.73	2.22 ± 2.81	Time	0.332	0.568	0.010
	T2	3.68 ± 3.73	2.04 ± 3.39	G * T	9.035	0.005	0.215
Verb. learn	T0	33.36 ± 8.64	37.35 ± 9.00	Group	5.071	0.031	1.33
	T1	31.84 ± 6.01	42.74 ± 10.81	Time	4.321	0.045	0.116
	T2	31.84 ± 6.01	45.83 ± 10.44	G * T	15.613	0.000	0.321
Reas/prob solving	T0	32.28 ± 4.71	34.43 ± 10	Group	17.056	0.000	0.341
	T1	30.08 ± 3.9	36.61 ± 8.59	Time	0.154	0.697	0.005
	T2	30.08 ± 3.09	39.35 ± 7.99	G * T	15.406	0.000	0.318
Visual learn	T0	35.92 ± 14.07	37.22 ± 15.68	Group	5.236	0.029	0.137
	T1	33.24 ± 12.19	45.09 ± 15.35	Time	0.095	0.760	0.003
	T2	33.24 ± 12.19	49.78 ± 13.58	G * T	4.208	0.048	0.113
Att/Vig	T0	28.36 ± 9.03	34.83 ± 10.57	Group	7.230	0.011	0.180
	T1	27.4 ± 7.27	36.43 ± 10.02	Time	1.799	0.189	0.052
	T2	27.4 ± 7.27	36.43 ± 12.06	G * T	0.504	0.483	0.015
SC	T0	27.4 ± 10.74	33.81 ± 12.53	Group	9.385	0.005	0.238
	T1	25.52 ± 5.9	38.67 ± 12.45	Time	3.333	0.78	0.100
	T2	25.52 ± 5.9	40.76 ± 12.48	G * T	6.710	0.015	0.183
Process speed	T0	24.48 ± 9.99	26.91 ± 7.72	Group	3.147	0.085	0.087
	T1	24.68 ± 7.11	32.48 ± 9.25	Time	0.439	0.512	0.013
	T2	24.68 ± 7.11	33 ± 11.12	G * T	5.491	0.025	0.143
Working memory	T0	29.24 ± 10.76	33.3 ± 8.57	Group	0.279	0.601	0.008
	T1	29.64 ± 11.16	37.04 ± 8.71	Time	1.261	0.270	0.037
	T2	29.64 ± 11.16	36.83 ± 10.7	G * T	0.863	0.360	0.025
SLOF total	T0	179.12 ± 17.36	177.7 ± 19.25	Group	0.247	0.622	0.007
	T1	175.84 ± 16.78	184.91 ± 12.81	Time	0.076	0.784	0.002
	T2	175.84 ± 16.77	184.43 ± 14.83	G * T	3.220	0.082	0.089
SLOF Pers. care	T0	32.76 ± 2.77	32.65 ± 3.3	Group	0.071	0.792	0.002
	T1	32.24 ± 2.49	33.35 ± 2.82	Time	0.812	0.374	0.024
	T2	32.24 ± 2.49	33.26 ± 2.03	G * T	0.484	0.492	0.014
SLOF Int. relation	T0	22.12 ± 4.3	20.96 ± 5.79	Group	1.146	0.292	0.34
	T1	20.8 ± 5.31	23.91 ± 4.33	Time	1.556	0.221	0.045
	T2	20.8 ± 5.31	24.04 ± 5.41	G * T	9.530	0.004	0.224
SLOF social acc	T0	31.44 ± 4.12	34.65 ± 0.78	Group	7.686	0.009	0.189
	T1	32.8 ± 3.86	34.83 ± 0.65	Time	0.306	0.584	0.009
	T2	32.8 ± 3.86	34.83 ± 0.39	G * T	0.709	0.406	0.021
SLOF activities	T0	47.76 ± 5.67	44.7 ± 6.87	Group	0.097	0.758	0.003
	T1	44.48 ± 6.97	46.13 ± 5.24	Time	0.006	0.938	0.000
	T2	44.98 ± 6.97	46.17 ± 5.93	G * T	3.885	0.049	0.105
SLOF work skills	T0	19.64 ± 5.37	19.74 ± 6.37	Group	0.071	0.792	0.002
	T1	20.32 ± 4.89	21.48 ± 5.6	Time	0.041	0.841	0.001
	T2	20.32 ± 4.89	21.61 ± 5.58	G * T	0.035	0.853	0.001
SUMD aw	T0	3.22 ± 0.65	2.74 ± 0.93	Group	3.654	0.065	0.100
	T1	3.22 ± 0.65	2.54 ± 0.72	Time	0.378	0.543	0.011
	T2	3.34 ± 0.66	2.49 ± 0.71	G * T	3.791	0.041	0.073
SUMD at	T0	3.35 ± 0.78	3.03 ± 1.13	Group	0.492	0.488	0.015
	T1	3.35 ± 0.78	2.97 ± 0.86	Time	0.113	0.739	0.003
	T2	3.43 ± 0.74	2.79 ± 0.88	G * T	0.005	0.944	0.000

*PANSS-P ± Positive and Negative Syndrome Scale ± positive symptoms; PANSS-N ± Positive and Negative Syndrome Scale ± negative symptoms; PANSS-G ± Positive and Negative Syndrome Scale ± general ± psychopathology; MAS Self-reflectivity ± Metacognition Assessment Scale ± self-reflectivity; MAS others' mind ± Metacognition Assessment Scale ± understanding of others' mind; MAS Decentrat ± Metacognition Assessment Scale ± decentration; MAS Mastery ± Metacognition Assessment Scale ± mastery; MAS Total ± Metacognition Assessment Scale ± total; CDSS ± Calgary Depression Symptom Scale; Verb. learn ± MCCB ± verbal learning; Reas/Prob solving ± MCCB ± reasoning and problem solving; Visual learn ± MCCB ± visual learning; Att/Vig ± MCCB ± attention/vigilance; SC ± MCCB ± social cognition; Process speed ± MCCB ± processing speed; Working memory ± MCCB ± working memory; SLOF Total ± Specific Levels of Functioning Scale ± total; SLOF Pers. care ± Specific Levels of Functioning Scale ± personal care skills; SLOF Int relation ± Specific Levels of Functioning Scale ± interpersonal relationships; SLOF Social acc ± Specific Levels of Functioning Scale ± social acceptability; SLOF Activities ± Specific Levels of Functioning Scale ± activities of community living; SLOF Work skill ± Specific Levels of Functioning Scale ± work skills; SUMD aw ± Scale to Assess Unawareness in Mental Disorder ± awareness; SUMD at ± Scale to Assess Unawareness in Mental Disorder ± attribution; CAG ± control active group; CACR ± computer-assisted cognitive remediation*.

## Discussion

Our study was aimed to assess the effectiveness of CACR vs. a CAG on specific cognitive, clinical, and functional domains in a sample of outpatients with SCZ. The CACR group was compared with an active experimental condition centered on non-specific elements of the remediation training, such as supportive therapist interactions and exposure to interesting computer activities. Outcomes were evaluated at three levels: proximally, on the remediation training exercises; intermediately, on neuropsychological measures not involved in the training; and more distally, on proxy measures of everyday functioning. Both groups were well-matched at baseline evaluation on demographic (except for age) and clinical confounding variables (i.e., metacognitive abilities) that might negatively impact outcome measures and might create a bias in study results. Furthermore, as indicated in a recent and relevant study, age does not appear to represent a significant moderator of effect (27).

There are several key observations from the study.

First, results showed a satisfactory adherence rate, with almost 70% of the participants completing the entire course of the protocol and adhering to over 80% of the prescribed sessions and outcome measures, suggesting that subjects did not seem to find the time commitment, the assessments, or the training burdensome. These completion rates are comparable with some studies (15, 28–30) and in contrast with other ones conducted so far (31), showing a higher attrition rate. Nevertheless, the present study adopted several measures to sustain adherence, mostly by strengthening the relationship with the therapist that has been shown to have a fundamental role in ensuring participants' completion of the training modules (32) and providing social cues to improve patients' self-esteem and motivation, supporting the use of strategies, motivation, or reinforcement and helping to develop metacognition, which is thought to be a key component for improving transfer from cognitive change to functional development (33, 34). Indeed, supportive, adaptive, and instructive trainers are thought to be a key ingredient of CACR programs and ultimately what makes them a therapeutic tool rather than a common “brain training,” being instrumental in achieving better training outcomes in CR (34, 35).

Second, while verbal learning and metacognitive sub-functions (understanding one's own mind and MAS total score) improved over assessment occasions, a significant time × group interaction was evident, suggesting that patients undergoing CACR intervention improved in domains related to specific cognitive domains (reasoning and problem solving, visual learning, verbal learning, and processing speed), understanding others' mind, mastery, SC, depressive symptoms, awareness of symptoms, and real-world functioning domains (community activities and interpersonal relationships) more significantly than did patients undergoing control active intervention.

Third, overall, the most noticeable differential improvement between the two groups were detected in two metacognitive sub-functions (understanding others' mind and mastery), in two cognitive domains (verbal learning and processing speed), in SLOF interpersonal relationship, in depressive symptomatology, and in SC, with effect sizes ranging from medium to high levels (see Table 1).

The positive findings regarding the amelioration of verbal and visual learning and reasoning and problem solving reported in the present study are consistent with the results of prior studies (13). The advantage of CR for verbal and visual learning support the possibility that at least some additional neurocognitive benefit may derive from an accurate titration of task difficulty of cognitive exercises to guarantee appropriate cognitive challenge, the rapid repetition of exacting exercises, and the frequency of reinforcement associated with achievement of intermediate and overall task goals characteristic of this condition. The hierarchical nature of the training program, starting with training in elementary attention skills and then gradually shifting to considerably more complex episodic and verbal memory tasks, might play a role in the advantage of this condition (36).

Thus, it is a paradox that there is no finding supporting an advantage of CACR on working memory (a skill related to holding information in mind and manipulating that information), while there is extensive evidence of CACR advantage in reasoning/executive-function and problem-solving domains. This result would not be expected because several studies have shown a close link between elementary working-memory functions and higher-level reasoning and problem-solving skills (37). Our results could be explained by the teaching strategies used by the therapist during and after CACR training sessions. Indeed, the one-to-one interaction with a therapist who can explicitly promote “bridging” strategies, as well as provide non-specific support and motivational coaching, represents important elements of the CACR therapy.

As for SC, we found a significant effect for this domain in the CACR group compared with the CAG. SC is the set of mental functions that allow individuals to interact with each other, i.e., the ability to understand, predict, and respond appropriately to the thoughts, feelings, and behavior of others in different and often unfamiliar social contexts (38, 39). The integrity of basic neuropsychological functions constitutes an essential prerequisite for the development of an adequate SC. Almost all published CACR studies agree that when coupled with rehabilitative programs focused on emotional intelligence, the benefits on recognition of emotions and the ability to interpret the feelings and behavior of others are improved (40, 41). However, the three studies included in the meta-analysis of Prikken et al. (13) did not detect a connection between CACR treatment and an improvement in emotional intelligence.

Alongside the improvement in neurocognitive functioning and SC obtained in the CACR group, we found an improvement in the metacognition (understanding others' mind and mastery) and insight (awareness of symptoms).

“Metacognition” identifies the main skills currently used to understand mental state and to properly attribute it to oneself or others. These skills include the ability to decipher the emotion expressions, to reason about mental states, and to use the obtained information to decide and solve problems and/or interpersonal conflicts. Finally, all these abilities also allow everyone to manage the subjective sufferance (42–44). It has been described as the memory processes that are related to the insights people have about their own and others' cognitive processes.

In order to develop an appropriate insight, metacognitive processes are fundamental because they allow individuals to notice changes in mental state over time and to produce plausible explanations for them, becoming more aware of their psychotic illness (45). Unfortunately, even if it is known that insight and metacognition are two integrative cognitive abilities, the exact neural and cognitive processes that support them are still unclear.

However, the great impairments in neurocognition (verbal, working, and visual memory; executive function; and overall intelligence) showed by patients with SCZ may be related to the severe deficits in metacognition (45–48). This impairment may lead to a lower ability to form complex mental representations of themselves and others (45, 46). In fact, SCZ patients with poor verbal memory might show more difficulties in remembering and integrating life experiences, even losing sense of previous experiences, with consequences in understanding the context complexity and their proper and others role on it (49).

Thus, the observed improvements in metacognitive skills and awareness of symptoms may be related to changes in neurocognitive functioning due to CACR in our study. Another possible explanation may be that the CACR group provided treatments in which patients' ability to reflect and to think about themselves was fostered to a greater extent than during a control active task. Indeed, all CACR sessions were facilitated by trained therapists who actively interacted with all participants to support their progression with the exercises, did troubleshooting, and spent time with participants on difficult exercises.

Moreover, participants showed significant improvements in two real-world functioning domains (interpersonal relationships and community activities) from baseline to 6-month follow-up with large effect sizes. The impact of CACR on psychosocial functioning is still controversial. A meta-analysis of Prikken et al. (13) found small-to-moderate but only marginally significant effects on functional outcomes. This finding is in contrast with other studies, also including strategy training, which showed larger and significant effects (9–11). It is plausible that CACR training alone might not be enough to improve daily functioning. Moreover, it has been observed that 6 months of treatment period might be too short to evaluate more differences in functioning between the two groups; probably, some changes in real-world functioning need a longer time to become evident, such as 1 year or more.

A plausible explanation of our results may be found on the fact that beneficial effects on verbal and visual memory may mediate the effect of CACR on social functioning; i.e., verbal memory has been considered to be associated with social competence (50) and daily functioning (51, 52); better visual–spatial memory after CACR may favor better skills in recognizing visual cues in social interactions and hence favor better social response and adaptation after CACR (53). Second, on the other hand, it appears that learning strategies could be a prerequisite for generalization of CACR treatment effects, explaining the reason why improvement in neurocognition does not automatically translate into improvement in social functioning (54, 55). Indeed, it was demonstrated that CACR can better influence functional outcomes when we give patients the opportunity to train cognitive skills in a context of a social learning environment through transferring skills from laboratory to real world (10, 55–57). According to this knowledge, our protocol study included a 10-min discussion with the therapist at the end of each CACR session in order to help in transferring learned abilities to the real world. Moreover, the differences in literature might depend on the different scales used to evaluate patients.

This study presents some limitations. First, the sample size is relatively small. Second, only patients in stable phase of disease were included, limiting generalization of the results toward the whole SCZ population. Third, there was no independent randomization. This clearly reduces methodological quality, although, current empirical evidence seems to indicate that non-randomized allocation does not influence effect sizes of cognitive training interventions (9). Fourth, the choice of the control intervention might represent another factor that theoretically has the potential to influence whether CACR is found to be effective or not (9). The CAG we employed engaged cognitive functions, not of a drill-and-practice type. A task with minimal demands on executive function was used, and while it does require memory skills, in this case, it involved procedural memory that is universally considered to be dissociable from episodic memory and trained in CACR (58). Furthermore, our control intervention therapeutic is unlikely to be considered because we found significant differences at the end of the treatment between the CACR and the CAG on some neuropsychological measures. Fifth, a factor that might have impacted the results of our study is that it was conducted in open units, where patients had the opportunity to go out to the community; this could have introduced uncontrolled variable, i.e., time spent in the community. Sixth, it could be argued that our study lacks enough power to detect differences between CACR and control group. It is a common issue in CACR trials, because detection of small effects requires large samples, and the recruitment of large group of patients is a great challenge, especially when trials are conducted in a single unit (59). Lastly, this study has not done a follow-up evaluation; for this reason, it lacks information about the duration of the outcomes.

Some strengths of this study deserve to be noticed, such as the well-matched baseline clinical, demographic, and cognitive characteristics of the two groups; the accurate assessment of cognitive functions, performed through the MCCB, and of real-world functioning, evaluated through the SLOF that is considered as the best scale to estimate psychosocial functioning of SCZ patients among those included in the VALERO program. Moreover, diagnoses were based on structured clinical interviews, and all patients were evaluated by trained raters. A further strength of this study is that the control group had the same number of hours of computer exposure as the treatment group. Lastly, another advantage is that there was no changing in pharmacological treatment during the study, allowing to specifically explain the effect of interventions.

In conclusion, if confirmed, our data point out the strength of CACR in implementing cognitive skills, SC, insight, metacognitive skills, and functioning in daily life in patients with SCZ. These are encouraging findings in support of the possible integration of CACR in rehabilitation practice in the Italian mental health services.

## Data Availability Statement

The raw data supporting the conclusions of this article will be made available by the authors, without undue reservation.

## Ethics Statement

The studies involving human participants were reviewed and approved by Comitato Etico Interaziendale, AOU Città della Salute e della Scienza, Torino. The patients/participants provided their written informed consent to participate in this study.

## Author Contributions

CM: conceptualization, methodology, investigation, and writing – original draft. ED and CR: conceptualization, investigation, and writing – original draft. LC, MT, and EZ: conceptualization and validation. PR: conceptualization, methodology, investigation, and formal analysis. All authors contributed to the article and approved the submitted version.

## Conflict of Interest

The authors declare that the research was conducted in the absence of any commercial or financial relationships that could be construed as a potential conflict of interest.

## References

[1] Allott K Liu P Proffitt TM Killackey E. Cognition at illness onset as a predictor of later functional outcome in early psychosis: systematic review and methodological critique. Schizophr Res. (2011) 125:221–35. 10.1016/j.schres.2010.11.001 21111577

[2] Bowie CR Leung WW Reichenberg A McClure MM Patterson TL Heaton RK. Predicting schizophrenia patients' real-world behavior with specific neuropsychological and functional capacity measures. Biol Psychiatry. (2008) 63:505–11. 10.1016/j.biopsych.2007.05.022 17662256 PMC2335305

[3] Green MF Kern RS Braff DL Mintz J. Neurocognitive deficits and functional outcome in schizophrenia: are we measuring the right stuff? Schizophr Bull. (2000) 26:119–36. 10.1093/oxfordjournals.schbul.a033430 10755673

[4] Seccomandi B Tsapekos D Newbery K Wykes T Cella M. A systematic review of moderators of cognitive remediation response for people with schizophrenia. Schizophr Res Cogn. (2019) 19:100160. 10.1016/j.scog.2019.100160 31828023 PMC6889639

[5] Bell MD Bryson G. Work rehabilitation in schizophrenia: does cognitive impairment limit improvement? Schizophr Bull. (2001) 27:269–79. 10.1093/oxfordjournals.schbul.a006873 11354594

[6] McGurk SR Meltzer HY. The role of cognition in vocational functioning in schizophrenia. Schizophr Res. (2000) 45:175–84. 10.1016/S0920-9964(99)00198-X 11042435

[7] Wykes T. Predicting symptomatic and behavioural outcomes of community care. Br J Psychiatry. (1994) 165:486–92. 10.1192/bjp.165.4.486 7804663

[8] Choi KH Wykes T Kurtz MM. Adjunctive pharmacotherapy for cognitive deficits in schizophrenia: meta-analytical investigation of efficacy. Br J Psychiatry. (2013) 203:172–8. 10.1192/bjp.bp.111.107359 23999481 PMC3759029

[9] Wykes T Huddy V Cellard C McGurk SR Czobor P. A meta-analysis of cognitive remediation for schizophrenia: methodology and effect sizes. Am J Psychiatry. (2011) 168:472–85. 10.1176/appi.ajp.2010.10060855 21406461

[10] McGurk SR Twamley EW Sitzer DI McHugo GJ Mueser KT. A meta-analysis of cognitive remediation in schizophrenia. Am J Psychiatry. (2007) 164:1791–802. 10.1176/appi.ajp.2007.07060906 18056233 PMC3634703

[11] Grynszpan O Perbal S Pelissolo A Fossati P Jouvent R Dubal S. Efficacy and specificity of computer-assisted cognitive remediation in schizophrenia: a meta-analytical study. Psychol Med. (2011) 41:163–73. 10.1017/S0033291710000607 20380784

[12] Revell ER Neill JC Harte M Khan Z Drake RJ. A systematic review and meta-analysis of cognitive remediation in early schizophrenia. Schizophr Res. (2015) 168:213–22. 10.1016/j.schres.2015.08.017 26305063

[13] Prikken M Konings MJ Lei WU Begemann MJH Sommer IEC. The efficacy of computerized cognitive drill and practice training for patients with a schizophrenia-spectrum disorder: a meta-analysis. Schizophr Res. (2019) 204:368–74. 10.1016/j.schres.2018.07.034 30097278

[14] Kambeitz-Ilankovic L Betz LT Dominke C Haas SS Subramaniam K Fisher M. Multi-outcome meta-analysis (MOMA) of cognitive remediation in schizophrenia: revisiting the relevance of human coaching and elucidating interplay between multiple outcomes. Neurosci Biobehav Rev. (2019) 107:828–45. 10.1016/j.neubiorev.2019.09.031 31557548 PMC8942567

[15] Bowie CR. Cognitive remediation for severe mental illness: state of the field and future directions. World Psychiatry. (2019) 18:274–5. 10.1002/wps.20660 31496075 PMC6732698

[16] Montemagni C Bracale N Bellino S Bozzatello P Enrico Z Toye M. Cognitive deficits and psychosocial functioning in schizophrenia: role of computer-assisted cognitive remediation. Evid Based Psychiatr Care. (2019) 5:1–9.

[17] Kay SR Fiszbein A Opler LA. The positive and negative syndrome scale (PANSS) for schizophrenia. Schizophr Bull. (1987) 13:261–76. 10.1093/schbul/13.2.261 3616518

[18] Addington D Addington J Schissel B. A depression rating scale for schizophrenics. Schizophr Res. (1990) 3:247–51. 10.1016/0920-9964(90)90005-R 2278986

[19] Amador XF Strauss DH. The Scale to assess Unawareness of Mental Disorder. New York, NY: Columbia University and New York Psychiatric Institute (1990).

[20] Nuechterlein KH Green MF Kern RS Baade LE Barch DM Cohen JD. The MATRICS consensus cognitive battery, part 1: test selection, reliability, and validity. Am J Psychiatry. (2008) 165:203–13 10.1176/appi.ajp.2007.07010042 18172019

[21] Mayer JD Salovey P Caruso D. Mayer-Salovey-Caruso Emotional Intelligence Test (MSCEIT) User's Manual. Toronto, ON: Multi-Health Systems. Inc. (2002).

[22] Semerari A Carcione A Dimaggio G Falcone M Nicolò G Procacci M. How to evaluate metacognitive functioning in psychotherapy? The metacognition assessment scale and its applications. Clin Psychol Psychother. (2003) 10:238–61. 10.1002/cpp.362

[23] Schneider LC Struening EL. SLOF: a behavioral rating scale for assessing the mentally ill. Soc Work Res Abstr. (1983) 19:9–21. 10.1093/swra/19.3.9 10264257

[24] Mucci A Rucci P Rocca P Bucci P Gibertoni D Merlotti E. Italian network for research on psychoses. The specific level of functioning scale: construct validity, internal consistency and factor structure in a large Italian sample of people with schizophrenia living in the community. Schizophr Res. (2014) 159:144–50. 10.1016/j.schres.2014.07.044 25182540

[25] Montemagni C Rocca P Mucci A Galderisi S Maj M. Italian version of the specific level of functioning. Versione italiana della specific level of functioning. J Psychopath. (2015) 21:287–96.

[26] Richardson JTE. Eta squared and partial eta squared as measures of effect size in educational research. Educ Res Rev. (2011) 6:135–47. 10.1016/j.edurev.2010.12.001

[27] Seccomandi B Agbedjro D Bell M Keefe RSE Keshavan M Galderisi S. Exploring the role of age as a moderator of cognitive remediation for people with schizophrenia. Schizophr Res. (2021) 228:29–35. 10.1016/j.schres.2020.11.060 33429151

[28] Klingberg S Wölwer W Engel C Wittorf A Herrlich J Meisner C. Negative symptoms of schizophrenia as primary target of cognitive behavioral therapy: results of the randomized clinical TONES study. Schizophr Bull. (2011) 37(Suppl 2):S98–110. 10.1093/schbul/sbr073 21860053 PMC3160126

[29] Wykes T Spaulding WD. Thinking about the future cognitive remediation therapy–what works and could we do better? Schizophr Bull. (2011) 37(Suppl 2):S80–90. 10.1093/schbul/sbr064 21860051 PMC3160118

[30] Siu AMH Ng RSH Poon MYC Chong CSY Siu CMW Lau SPK. Evaluation of a computer-assisted cognitive remediation program for young people with psychosis: a pilot study. Schizophr Res Cogn. (2020) 23:100188. 10.1016/j.scog.2020.100188 32983917 PMC7493079

[31] Dillon R Hargreaves A Anderson-Schmidt H Castorina M Corvin A Fitzmaurice B. Adherence to a low-support cognitive remediation training program for psychosis. J Nerv Ment Dis. (2016) 204:741–5. 10.1097/NMD.0000000000000557 27385473

[32] Garrido G Barrios M Penadés R Enríquez M Garolera M Aragay N. Computer-assisted cognitive remediation therapy: cognition, self-esteem and quality of life in schizophrenia. Schizophr Res. (2013) 150:563–9. 10.1016/j.schres.2013.08.025 24035402

[33] Cella M Reeder C Wykes T. Cognitive remediation in schizophrenia—Now it is really getting personal. Curr Opin Behav Sci. (2015) 4:147–51. 10.1016/j.cobeha.2015.05.005

[34] Contreras NA Lee S Tan EJ Castle DJ Rossell SL. How is cognitive remediation training perceived by people with schizophrenia? A qualitative study examining personal experiences. J Ment Health. (2016) 25:260–6. 10.3109/09638237.2016.1167856 27045420

[35] Moura BM Avila A Chendo I Frade P Barandas R Vian J. Facilitating the delivery of cognitive remediation in first-episode psychosis: pilot study of a home-delivered web-based intervention. J Nerv Ment Dis. (2019) 207:951–7. 10.1097/NMD.0000000000001055 31503184

[36] Kurtz MM Seltzer JC Shagan DS Thime WR Wexler BE. Computer-assisted cognitive remediation in schizophrenia: what is the active ingredient? Schizophr Res. (2007) 89:251–60. 10.1016/j.schres.2006.09.001 17070671 PMC2095777

[37] Gold JM Carpenter C Randolph C Goldberg TE Weinberger DR. Auditory working memory and Wisconsin Card Sorting Test performance in schizophrenia. Arch Gen Psychiatry. (1997) 54:159–65. 10.1001/archpsyc.1997.01830140071013 9040284

[38] Hogarty G Flesher S. Developmental theory for a cognitive enhancement therapy of schizophrenia. Schizophr Res. (1999) 25:677–92. 10.1093/oxfordjournals.schbul.a033410 10667739

[39] Frith CD Frith U. Social cognition in humans. Curr Biol. (2007) 17:R724–32. 10.1016/j.cub.2007.05.068 17714666

[40] Wölwer W Frommann N Halfmann S Piaszek A Streit M Gaebel W. Remediation of impairments in facial affect recognition in schizophrenia: efficacy and specificity of a new training program. Schizophr Res. (2005) 80:295–303. 10.1016/j.schres.2005.07.018 16125367

[41] Lindenmayer JP McGurk SR Khan A Kaushik S Thanju A Hoffman L. Improving social cognition in schizophrenia: a pilot intervention combining computerized social cognition training with cognitive remediation. Schizophr Bull. (2013) 39:507–17. 10.1093/schbul/sbs120 23125396 PMC3627756

[42] Carcione A Nicolò G Pedone R Popolo R Conti L Fiore D. Metacognitive mastery dysfunctions in personality disorder psychotherapy. Psychiatry Res. (2011) 190:60–71. (2010) 12.032. 10.1016/j.psychres.2010.12.032 21329989

[43] Brüne M Abdel-Hamid M Lehmkämper C Sonntag C. Mental state attribution, neurocognitive functioning, and psychopathology: what predicts poor social competence in schizophrenia best? Schizophr Res. (2007) 92:151–9. 10.1016/j.schres.2007.01.006 17346931

[44] Tas C Brown EC Aydemir O Brüne M Lysaker PH. Metacognition in psychosis: comparison of schizophrenia with bipolar disorder. Psychiatry Res. (2014) 219:464–9. 10.1016/j.psychres.2014.06.040 25017619

[45] Lysaker PH Carcione A Dimaggio G Johannesen JK Nicolò G Procacci M. Metacognition amidst narratives of self and illness in schizophrenia: associations with neurocognition, symptoms, insight and quality of life. Acta Psychiatr Scand. (2005) 112:64–71. 10.1111/j.1600-0447.2005.00514.x 15952947

[46] Nicolò G Dimaggio G Popolo R Carcione A Procacci M Hamm J. Associations of metacognition with symptoms, insight, and neurocognition in clinically stable outpatients with schizophrenia. J Nerv Ment Dis. (2012) 200:644–7. 10.1097/NMD.0b013e31825bfb10 22759945

[47] Brune M. Theory of mind and the role of IQ in chronic disorganized schizophrenia. Schizophr Res. (2003) 60:57–64. 10.1016/S0920-9964(02)00162-7 12505138

[48] Doody GA Götz M Johnstone EC Frith CD Owens DG. Theory of mind and psychoses. Psychol Med. (1998) 28:397–405. 10.1017/S003329179700648X 9572096

[49] Leonhardt BL Vohs JL Bartolomeo LA Visco A Hetrick WP Bolbecker AR. Relationship of metacognition and insight to neural synchronization and cognitive function in early phase psychosis. Clin EEG Neurosci. (2020) 51:259–66. 10.1177/1550059419857971 31241355

[50] McClure MM Bowie CR Patterson TL Heaton RK Weaver C Anderson H. Correlations of functional capacity and neuropsychological performance in older patients with schizophrenia: evidence for specificity of relationships? Schizophr Res. (2007) 89:330–8 10.1016/j.schres.2006.07.024 16982175

[51] Penadés R Catalán R Puig O Masana G Pujol N Navarro V. Executive function needs to be targeted to improve social functioning with Cognitive Remediation Therapy (CRT) in schizophrenia. Psychiatry Res. (2010) 177:41–5. 10.1016/j.psychres.2009.01.032 20381164

[52] Lanfredi M Deste G Ferrari C Barlati S Magni LR Rossi R. Effects of cognitive remediation therapy on neurocognition and negative symptoms in schizophrenia: an Italian naturalistic study. Cogn Neuropsychiatry. (2017) 22:53–68. 10.1080/13546805.2016.1260537 27921860

[53] Lindenmayer JP Fregenti S Kang G Ozog V Ljuri I Khan A. The relationship of cognitive improvement after cognitive remediation with social functioning in patients with schizophrenia and severe cognitive deficits. Schizophr Res. (2017) 185:154–60. 10.1016/j.schres.2017.01.007 28094171

[54] Lu H Li Y Li F Jiao X Shi W Guo K. Randomized controlled trial on adjunctive cognitive remediation therapy for chronically hospitalized patients with schizophrenia. Shanghai Arch Psychiatry. (2012) 24:149–54. 10.3969/j.issn.1002-0829.2012.03.004 25324619 PMC4198846

[55] Medalia A Saperstein AM. Does cognitive remediation for schizophrenia improve functional outcomes? Curr Opin Psychiatry. (2013) 26:151–7. 10.1097/YCO.0b013e32835dcbd4 23318663

[56] Nibbio G Barlati S Cacciani P Corsini P Mosca A Ceraso A. Evidence-based integrated intervention in patients with schizophrenia: a pilot study of feasibility and effectiveness in a real-world rehabilitation setting. Int J Environ Res Public Health. (2020) 17:3352. 10.3390/ijerph17103352 32408561 PMC7277196

[57] Van Duin D de Winter L Oud M Kroon H Veling W van Weeghel J. The effect of rehabilitation combined with cognitive remediation on functioning in persons with severe mental illness: systematic review and meta-analysis. Psychol Med. (2019) 49:1414–25. 10.1017/S003329171800418X 30696500

[58] Gomar JJ Valls E Radua J Mareca C Tristany J del Olmo F. Cognitive rehabilitation study group. A multisite, randomized controlled clinical trial of computerized cognitive remediation therapy for schizophrenia. Schizophr Bull. (2015) 41:1387–96. 10.1093/schbul/sbv059 26006264 PMC4601710

[59] Linke M Jankowski KS Wichniak A Jarema M Wykes T. Effects of cognitive remediation therapy versus other interventions on cognitive functioning in schizophrenia inpatients. Neuropsychol Rehabil. (2019) 29:477–88. 10.1080/09602011.2017.1317641 28457189

